# Factors associated with loss to follow up after entry into care of HIV infected children ineligible for antiretroviral therapy: data from an HIV cohort study in India

**DOI:** 10.1186/1471-2334-14-S3-O26

**Published:** 2014-05-27

**Authors:** Praveen Kumar Naik, Manoranjan Midde, Raghavakalyan Pakam, Gerardo Alvarez-Uria

**Affiliations:** 1 Department of Infectious Diseases, Rural Development Trust Hospital, Bathalapalli, AP, India

## Background

Half of HIV-infected adults who are not eligible for antiretroviral therapy (ART) are lost to follow up (LTFU). However, data about the attrition from enrolment in care to ART eligibility of HIV infected children are scarce.

## Methods

Two hundred and eighty two children ineligible for ART at enrolment in care were followed up until ART eligibility. Multivariable analysis was performed using competing risk regression. The 12-month risk of AIDS was calculated using the age and the absolute CD4 cell count as in the HIV Pediatric Prognostic Markers Collaborative Study.

## Results

The cumulative incidence of attrition (mortality and LTFU) was 15.6% (95% confidence interval [CI], 11.3-20.5) after six years of follow-up, and the attrition rate was higher during the first year after enrolment. Children with a 12-month risk of AIDS < 3% had a higher risk of LTFU (subdistribution hazard ratio [SHR] 10.77, 95% CI 1.93-60.07) than those with a 12-month risk of AIDS >4%. Those children whose father had died had a lower risk of LTFU (SHR 0.26, 95% CI 0.09-0.75) than those whose parents were alive and were living in a rented house. Children aged 10-14 had a lower risk of LTFU (SHR 0.12, 95% CI 0.03-0.55) than those aged 5-9 years.

## Conclusions

A substantial proportion of children ineligible for ART were LTFU before ART eligibility. These findings can be used by HIV programmes to design interventions aimed at reducing the attrition in pre-ART care of HIV infected children in India.

